# Rivaroxaban versus Apixaban for Treatment of Cancer-Associated Venous Thromboembolism in Patients at Lower Risk of Bleeding

**DOI:** 10.1055/s-0043-1770783

**Published:** 2023-07-10

**Authors:** Kimberly Snow Caroti, Cecilia Becattini, Marc Carrier, Alexander T. Cohen, Anders Ekbom, Alok A. Khorana, Agnes Y.Y. Lee, Christopher Brescia, Khaled Abdelgawwad, George Psaroudakis, Marcela Rivera, Bernhard Schaefer, Gunnar Brobert, Craig I. Coleman

**Affiliations:** 1 Department of Pharmacy Practice, School of Pharmacy, University of Connecticut, Storrs, Connecticut, United States; 2 Evidence-Based Practice Center, Hartford Hospital, Hartford, Connecticut, United States; 3 Department of Internal and Emergency Medicine – Stroke Unit, University of Perugia, Perugia, Italy; 4 Department of Medicine, Ottawa Hospital Research Institute at the University of Ottawa, Ottawa, Canada; 5 Department of Haematological Medicine, Guy's and St Thomas' NHS Foundation Trust, King's College London, London, United Kingdom; 6 Unit of Clinical Epidemiology, Department of Medicine, Karolinska Institute, Stockholm, Sweden; 7 Cleveland Clinic and Case Comprehensive Cancer Center, Cleveland, Ohio, United States; 8 Department of Medicine, University of British Columbia and BC Cancer, Vancouver, Canada; 9 Freshtech IT, LLC, East Hartford, Connecticut, United States; 10 Pharmacoepidemiology Group, Bayer AG, Berlin, Germany

**Keywords:** apixaban, rivaroxaban, venous thromboembolism, cancer-associated VTE

## Abstract

This retrospective study, utilizing U.S. electronic health record (EHR) data from January 2013 to December 2020, sought to assess whether rivaroxaban and apixaban had similar effectiveness and safety in the treatment of cancer-associated venous thromboembolism (VTE) in patients with a cancer type not associated with a high risk of bleeding. We included adults diagnosed with active cancer, excluding esophageal, gastric, unresected colorectal, bladder, noncerebral central nervous system cancers and leukemia, who experienced VTE and received a therapeutic VTE dose of rivaroxaban or apixaban on day 7 post-VTE, and were active in the EHR ≥12 months prior to the VTE. Primary outcome was the composite of recurrent VTE or any bleed resulting in hospitalization at 3 months. Secondary outcomes included recurrent VTE, any bleed resulting in hospitalization, any critical organ bleed, and composites of these outcomes at 3 and 6 months. Inverse probability of treatment-weighted Cox regression was used to calculate hazard ratios (HRs) with 95% confidence intervals (CIs). We included 1,344 apixaban and 1,093 rivaroxaban patients. At 3 months, rivaroxaban was found to have similar hazard to apixaban for developing recurrent VTE or any bleed resulting in hospitalization (HR: 0.87; 95% CI: 0.60–1.27). No differences were observed between cohorts for this outcome at 6 months (HR: 1.00; 95% CI: 0.71–1.40) or for any other outcome at 3 or 6 months. In conclusion, patients receiving rivaroxaban or apixaban showed similar risks of the composite of recurrent VTE or any bleed resulting in hospitalization in patients with cancer-associated VTE. This study was registered at www.clinicaltrials.gov as #NCT05461807.

**Key Points**

Rivaroxaban and apixaban have similar effectiveness and safety for treatment of cancer-associated VTE through 6 months.

Clinicians should therefore consider patient preference and adherence when choosing the optimal anticoagulant.

## Graphical abstract

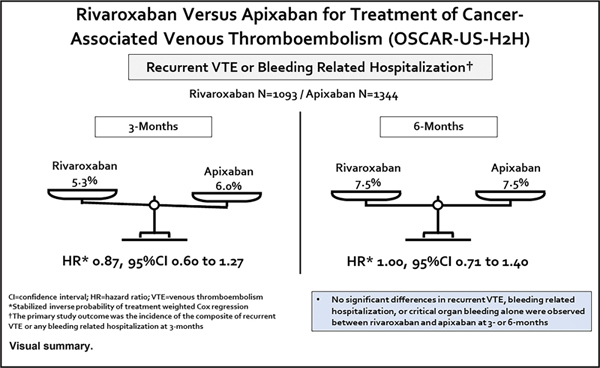

## Introduction

Patients with active cancer are at as high as 12-fold more likely to develop a venous thromboembolism (VTE) than those without. 1 When VTE occurs, patients with cancer carry up to a threefold higher rate of thrombosis recurrence and approximately twice the risk of bleeding during anticoagulation. 1 2 3 Therefore, it is critical to utilize anticoagulants that optimize effectiveness while minimizing bleeding risk when treating cancer-associated VTE (Ca-VTE). The strength of recommendation in Ca-VTE guidelines 4 5 6 7 8 9 for oral factor Xa inhibitors is based on data from randomized controlled trials (RCTs) 10 11 12 13 14 15 comparing an oral factor Xa inhibitor to low-molecular-weight heparins (LMWHs) to treat Ca-VTE. Results of these RCTs suggest that oral factor Xa inhibitors may reduce thrombosis risk but with potentially more frequent bleeding; the latter finding was disproportionally driven by patients with gastrointestinal and genitourinary malignancies. 10 No head-to-head RCT of rivaroxaban versus apixaban in the treatment of Ca-VTE is available. Therefore, we sought to evaluate the effectiveness and safety of rivaroxaban versus apixaban for Ca-VTE treatment in a retrospective cohort of patients with active cancer considered at low risk of bleeding. 4 5 6 7 8 9

## Methods

### Data Source

The Head-to-Head Observational Study in Cancer Associated Thrombosis for Rivaroxaban—United States Cohort (H2H-OSCAR-US) protocol was registered on ClinicalTrials.gov Identifier (NCT05461807). We performed a retrospective cohort analysis using U.S. Optum De-Identified electronic health record (EHR) data from January 1, 2012 through December 31, 2020. The Optum EHR database provided longitudinal patient-level medical record data for >95 million patients seen at approximately 700+ hospitals and approximately 7,000+ clinics across the United States and includes data on medications both electronically prescribed and self-reported (including over-the-counter medications), laboratory results, vital signs, body measurements, other clinical observations, and diagnosis and procedure codes. 11 Insured and uninsured patients of all ages are included in the data set. The use of the Optum EHR database does not involve human subjects research and has been determined by the New England Institutional Review Board to be exempt from broad institutional review board approval. All Optum EHR data are deidentified and follow the Health Insurance Portability and Accountability Act of 1996 to preserve patient anonymity and confidentiality.

### Cohort Selection

The population of interest for this study included adult patients with active cancer excluding esophageal, gastric, unresected colorectal, bladder, noncerebral central nervous system cancers, and leukemia, 4 5 6 7 8 9 who were admitted to the hospital, emergency department, or observation unit for acute deep vein thrombosis (DVT) and/or pulmonary embolism (PE) on or after January 1, 2013, and were treated with therapeutic VTE doses of rivaroxaban or apixaban per electronically entered prescription or patient self-report on day 7 after the qualifying acute VTE diagnosis (index date). Patients had to be active in the EHR data set ≥12 months prior to the index event and had ≥1 provider visit in the 12 months prior to the acute VTE event (baseline period). We defined active cancer as cancer being actively treated with systemic therapy or surgery within 6 months of the index Ca-VTE, or metastatic disease regardless of time from initial cancer diagnosis or treatment. We excluded patients if they had an alternative indication for anticoagulation, were anticoagulated in the 12 months prior, or were pregnant.

### Outcomes

We assessed the primary composite of recurrent VTE (defined by the presence of an appropriate inpatient discharge diagnosis code in the primary coding position) 12 or any bleed resulting in hospitalization (defined per the Cunningham algorithm 13 ) at 3 months. Secondary outcomes included a composite of recurrent VTE or any critical organ bleed (defined as intracranial, intraspinal, intraocular, retroperitoneal, intraarticular, pericardial, or intramuscular with compartment syndrome); recurrent VTE; any bleed resulting in hospitalization; and any critical organ bleed at 3 and at 6 months. To evaluate if residual confounding bias might have influenced the results, we assessed hospitalization for any pneumonia as a falsification outcome (e.g., an outcome that is not anticipated to be impacted by anticoagulant choice and thus should demonstrate a null effect in the absence of residual confounding). 14

### Sample Size

This study was powered to demonstrate the choice of rivaroxaban versus apixaban in the management of Ca-VTE would not result in an excessive increase in the composite outcome of recurrent VTE or any bleed resulting in hospitalization at 3 months. We hypothesized that rivaroxaban would have similar effectiveness and safety to apixaban with respect to the risk of recurrent VTE or any bleed resulting in hospitalization, defined as an upper limit of the 95% confidence interval (CI) for the hazard ratio (HR) of <1.50 15 and a one-sided alpha level of 0.025. 15 Assuming a 3-month risk of the primary outcome of 7% 15 16 and no difference between rivaroxaban and apixaban (i.e., HR: 1.0), we estimated a sample of approximately 1,900 patients would be required to observe an expected total of 100 primary outcome events and would give our study 80% power. Based on previous analyses of this Optum EHR dataset, we anticipated approximately 6,000 patients experiencing Ca-VTE, of which, >1,000 patients would have received rivaroxaban and >1,000 would have received apixaban. 16

### Statistical Analysis

To adjust for potential confounding between the rivaroxaban and apixaban cohorts, propensity scores were calculated using a multivariable logistic regression model. 17 The propensity score model utilized 67 covariates, including demographics, baseline comorbidities, laboratory values, vital signs, other clinical observations, systemic cancer treatments and surgeries, and medication use (electronic prescription and over-the-counter). All clinical characteristics listed in Table 1 were included in the propensity score model. Propensity scores were then used to assign weights to individual patients in the analysis using a stabilized inverse probability of treatment weighting (IPTW) approach. Adequacy of weighting was assessed by reviewing absolute standardized differences (ASDs) for all covariates entered in the propensity score model. An ASD <0.10 was considered to represent adequate balance. 17

**Table 1:** Characteristics of unweighted and stabilized inverse probability treatment-weighted patient demographics of rivaroxaban and apixaban patients with cancer-associated VTE

	Unweighted				sIPTW		
	All, *N* = 2,437, %	Rivaroxaban, *n* = 1,093, %	Apixaban, *n* = 1,344, %	ASD	Rivaroxaban, *n* = 1,093, %	Apixaban, *n* = 1,344, %	ASD
Aged ≤40 y	3.1	3.8	2.6	0.06	3.2	3.2	0.00
Aged 41–60 y	27.6	31.2	24.7	0.14	26.8	26.6	0.00
Aged 61–74 y	40.8	40.4	41.2	0.01	41.3	41.0	0.00
Aged ≥75 y	28.4	24.6	31.6	0.16	28.7	29.3	0.00
BMI ≤29 kg/m ^2^	56.3	55.7	56.7	0.02	55.2	56.3	0.00
BMI 30–34 kg/m ^2^	23.1	23.4	22.9	0.01	24.3	23.0	0.01
BMI 35–39 kg/m ^2^	10.3	11.3	9.5	0.06	9.9	10.2	0.01
BMI ≥40 kg/m ^2^	10.3	9.5	10.9	0.05	10.6	10.6	0.00
Female	58.1	58.8	57.5	0.03	56.3	57.4	0.00
January 1, 2013 to anticoagulation, mean ± SD, d	1,932 ± 679	1,617 ± 711	2,188 ± 529	0.80	1,955 ± 698	2,002 ± 588	0.07
Number of hospitalizations in previous 12 months ≥2	44.6	39.6	48.7	0.18	42.9	44.4	0.01
Active cancer treatment within 4 weeks	57.2	54.2	59.8	0.11	57.8	57.8	0.00
Metastatic disease	37.3	36.7	37.8	0.02	37.5	37.9	0.00
eGFR <30 mL/min	5.1	4.5	5.7	0.06	5.9	5.2	0.03
eGFR 30–59 mL/min	13.2	11.3	14.7	0.11	12.9	13.3	0.01
eGFR 60–89 mL/min	43.4	42.4	44.3	0.04	43.1	43.5	0.01
eGFR >90 mL/min	38.3	42.0	35.3	0.13	38.1	38.1	0.00
Chronic lung disease	31.6	29.7	33.0	0.07	31.3	31.6	0.01
Rheumatic disease	6.1	6.0	6.3	0.01	6.0	6.5	0.02
Liver disease	5.7	6.4	5.2	0.05	5.5	5.1	0.02
Heart failure	9.2	8.2	9.9	0.06	9.3	9.3	0.00
Stroke or systemic embolism	5.0	3.8	6.1	0.12	4.6	4.9	0.01
Prior myocardial infarction	6.6	5.3	7.7	0.11	5.7	6.8	0.05
Peripheral arterial disease	7.9	7.3	8.3	0.04	7.8	8.3	0.02
Hypertension	65.7	60.7	68.8	0.17	67.0	66.4	0.01
Varicose veins	2.3	2.0	2.6	0.04	2.4	2.3	0.01
Any prior bleed	7.6	8.3	7.1	0.05	7.5	7.6	0.00
PE ± DVT	46.8	42.0	50.7	0.18	45.0	46.1	0.02
Prior history of VTE	11.0	12.0	10.3	0.05	10.5	10.4	0.00
Frailty score ≥16	12.8	10.2	14.9	0.16	13.9	13.1	0.02
Coagulopathy	12.8	9.9	15.1	0.18	12.8	13.0	0.01
Impaired mobility	2.1	1.6	2.5	0.07	1.7	2.1	0.03
P2Y _12_ inhibitor	2.5	1.8	3.0	0.09	2.0	2.3	0.02
Aspirin	17.6	17.5	17.7	0.01	17.4	17.6	0.01
NSAID	42.6	43.4	42.0	0.03	43.2	42.6	0.01
Statin	39.0	35.6	41.7	0.13	39.3	39.1	0.00
PPI or H2 receptor antagonist	56.9	54.1	59.2	0.10	56.8	58.0	0.02
Estrogen	3.0	3.6	2.5	0.06	2.7	2.9	0.01
Intravenous/subcutaneous anticoagulant first	61.3	65.4	58.0	0.16	61.8	61.1	0.01
Laboratory values							
Alkaline phosphatase, mean ± SD, U/L	108.6 ± 100.3	108.6 ± 104.1	108.7 ± 97.1	0.00	108.3 ± 96.8	107.9 ± 96.6	0.00
Hemoglobin, mean ± SD, g/dL	11.8 ± 2.1	11.9 ± 2.0	11.7 ± 2.1	0.14	11.8 ± 2.0	11.8 ± 2.1	0.00
Platelets, mean ± SD, × 10 ^3^ /µL	238.7 ± 103.9	242.2 ± 102.4	235.8 ± 105.0	0.06	239.2 ± 102.4	239.8 ± 107.4	0.01
Total bilirubin, mean ± SD, mg/dL	0.6 ± 0.9	0.7 ± 0.7	0.6 ± 1.0	0.02	0.6 ± 0.6	0.6 ± 1.0	0.00
Absolute neutrophil count, <1500 cells/µL	3.8	3.2	4.2	0.06	3.7	3.7	0.00
Serum albumin, mean ± SD, g/dL	3.5 ± 0.6	3.5 ± 0.6	3.5 ± 0.6	0.09	3.5 ± 0.6	3.5 ± 0.6	0.00
Cancer type							
Resected colorectal	1.3	1.5	1.0	0.04	1.2	1.1	0.01
Lung	19.8	18.3	21.0	0.07	19.7	20.6	0.02
Ovarian	3.6	3.8	3.4	0.02	3.2	3.5	0.02
Brain	2.6	2.4	2.8	0.03	3.1	2.8	0.02
Urologic	4.8	4.9	4.8	0.00	4.9	4.8	0.00
Hepatobiliary	12.0	11.2	12.7	0.05	12.0	12.1	0.00
Breast	24.3	25.3	23.4	0.04	23.8	23.2	0.01
Gynecologic	9.8	9.9	9.8	0.00	9.3	9.8	0.02
Pancreatic	4.3	3.6	5.0	0.08	4.1	4.3	0.01
Lymphoma	8.4	9.4	7.6	0.06	8.6	8.5	0.00
Prostate	13.9	13.0	14.6	0.05	15.0	14.1	0.03
Kidney	4.5	4.5	4.5	0.00	4.6	4.5	0.00
Myeloma	3.4	3.3	3.4	0.01	3.2	3.4	0.01
Testicular	0.8	0.6	0.9	0.03	0.6	0.7	0.01
Other	15.7	17.3	14.4	0.08	15.7	15.7	0.00
Systemic cancer therapies							
Hormonal therapy	19.5	20.6	18.5	0.05	18.7	18.6	0.00
Kinase inhibitors	3.7	3.8	3.6	0.01	3.7	3.3	0.02
Monoclonal antibodies	2.6	2.5	2.7	0.01	2.2	2.5	0.02
Immunomodulating agents	0.3	0.1	0.5	0.12	0.2	0.3	0.02
Miscellaneous	1.0	1.1	0.9	0.02	1.0	0.9	0.01
Antimetabolites	6.2	6.4	6.0	0.02	6.5	6.2	0.01
Alkylating agents	3.4	3.4	3.4	0.00	3.8	3.6	0.01
Antitumor antibiotics	0.3	0.4	0.2	0.02	0.2	0.3	0.02
Proteasome inhibitors	1.2	1.0	1.3	0.03	1.0	1.2	0.02
Platinum-based chemotherapy	4.3	4.1	4.4	0.01	4.6	4.3	0.01
Anthracyclines	0.0	0.0	0.1	0.04	0.0	0.1	0.04
Topoisomerase inhibitors	1.1	1.2	1.0	0.01	1.0	1.0	0.00
Vinca alkaloids	0.4	0.2	0.5	0.08	0.4	0.5	0.01
Bevacizumab	0.0	0.0	0.0	0.00	0.0	0.0	0.00
Taxanes	3.4	3.3	3.4	0.01	4.2	3.6	0.03

Abbreviations: ASD, absolute standardized difference; BMI, body mass index; DVT, deep vein thrombosis; eGFR, estimated glomerular filtration rate; H2, histamine type-2; NSAID, nonsteroidal anti-inflammatory drug; PE, pulmonary embolism; PPI, proton pump inhibitor; sIPTW, stabilized inverse probability of treatment weighting; VTE, venous thromboembolism.

Given the retrospective nature of the data analysis, the presence of a comorbid disease diagnosis was made based upon billing codes and/or supporting laboratory and observation data. The absence of data for a comorbidity was assumed to represent the absence of the disease (thus no missing data for binary comorbidity disease diagnoses). For continuous laboratory values and observations data, missing data were imputed using a multiple imputation approach based on a fully conditional specification linear regression model with all other available variables included in the model. No imputation was performed for missing outcomes data.

Baseline characteristics were analyzed using descriptive statistics for unweighted and stabilized IPTW cohorts. 17 Categorical data were reported as percentages and continuous data as medians with accompanying interquartile ranges or means ± standard deviations. Kaplan–Meier analysis was performed to generate time-to-event curves. We fit Cox proportional hazards regression models with robust sandwich estimators to compare event rates over time for rivaroxaban versus apixaban for all outcomes. The only independent variable included into Cox regression models was anticoagulant received. Results of Cox regression analyses were reported as HRs with 95% CIs. Data management and statistical analysis was performed using SAS version 9.4 (SAS Institute, Cary, North Carolina) and IBM SPSS version 28.0 (IBM Corp., Armonk, New York). The proportional hazard assumption was tested based on Schoenfeld residuals and found to be met for all outcomes. Patients were censored in the Cox models at the first incidence a patient experienced end-of-EHR activity (based on “Last Month Active” data available in the Optum EHR) or reached the end of data availability in the Optum data set. Due to lack of prescription fill claims data in the data set, 11 it was anticipated that we would be unable to accurately assess patient time on anticoagulation for a substantial proportion of the study population. Patients were therefore analyzed using an intent-to-treat approach (whereby patients were evaluated based on their anticoagulant received on day 7 and were not censored at therapy switch or discontinuation). Time from treatment initiation to end of follow-up was considered the time under risk. A *p* -value <0.05 was considered significant in all cases. No adjustments for multiple hypothesis testing were performed.

### Research Reporting

This article was written in accordance with the reporting of studies conducted using observational routinely collected health data statement for pharmacoepidemiology guidance. 18

### Role of Funding Source

This study was supported by Bayer AG, Berlin, Germany. The corresponding author had full access to all the data in the study and had final responsibility for the decision to submit for publication.

### Data Sharing Statement

Data used in this study were obtained from Optum under a license to Bayer AG (and provided to Dr. Coleman under a third-party agreement) and are not publicly available.

## Results

### Patient Characteristics

**Fig. 1:**
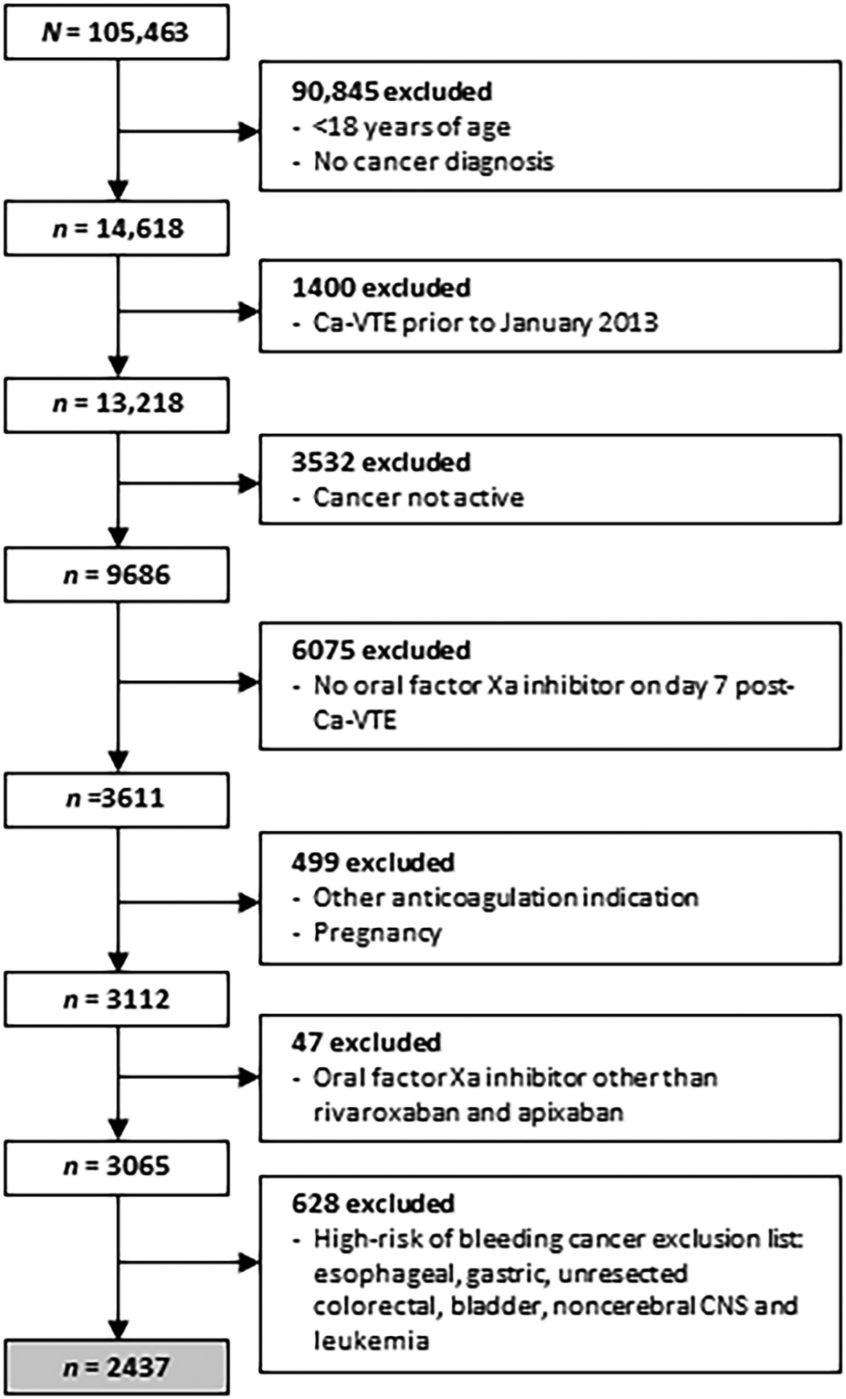
Patient inclusion and exclusion from the study. Of 14,618 patients with Ca-VTE initially identified, 12,181 were excluded, resulting in a final study population of 2,437 patients with cancer types for which oral factor Xa inhibitors are recommended according to guidelines and receiving either rivaroxaban or apixaban for inclusion in the analysis. Ca-VTE, cancer-associated venous thromboembolism; CNS, central nervous system.

The EHR data set included 105,463 patients who had a primary hospital, emergency department, or observation unit billing code for VTE. Of these, 12.5% were adult patients with a diagnosis of cancer and had their VTE on or after January 1, 2013. Approximately 27% of these patients lacked evidence of active cancer (cancer treatment within 6 months or metastatic disease). Additional patients were excluded from the analysis because they were not receiving rivaroxaban or apixaban on day 7 post-Ca-VTE diagnosis, had an alternative indication for full-dose anticoagulation, or were pregnant. This left a total of 2,437 patients with active cancer experiencing a Ca-VTE and treated with either rivaroxaban ( *n* = 1,093) or apixaban ( *n* = 1,344) available for analysis ( Fig. 1 ).

After IPTW, characteristics of patients receiving rivaroxaban and apixaban were similar for all covariates included in the propensity score model ( Table 1 ). Of included patients, 29.0% were ≥75 years of age, 56.9% were female, 20.6% had a body mass index (BMI) ≥35 kg/m ^2^ , and 18.7% had an estimated glomerular filtration rate (eGFR) <60 mL/minute at baseline. The Ca-VTE event was a PE ± DVT in 45.6% of patients, 37.7% had metastatic disease, and 46.9% received active cancer treatment within 4 weeks of the Ca-VTE event. The most common cancer types (>10% prevalence) included breast (23.5%), lung (20.1%), prostate (14.6%), and hepatobiliary (12.1%).

### Main Results

**Fig. 2:**
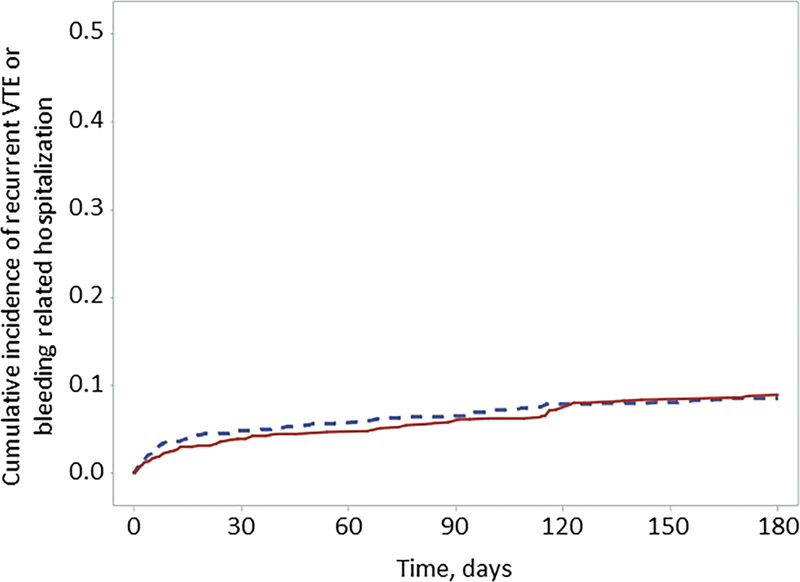
Time to recurrent VTE or bleeding-related hospitalization in rivaroxaban and apixaban patients. Kaplan–Meier curve for the composite of recurrent VTE or bleeding-related hospitalization (rivaroxaban = solid line, apixaban = dashed line). VTE, venous thromboembolism.

At 3 months, rivaroxaban and apixaban were found to be associated with a similar hazard of developing the composite of recurrent VTE or any bleed resulting in hospitalization (5.3 vs. 6.0% for rivaroxaban and apixaban [referent], respectively; HR: 0.87, 95% CI: 0.60–1.27) ( Fig. 2 , Table 2 ). No significant differences were observed between anticoagulation cohorts for this outcome at 6 months (HR: 1.00, 95% CI: 0.71–1.40) or for any other outcome at 3 or 6 months ( Supplementary Figs. S1–S4 , available in the online version).

**Table 2:** Outcomes of stabilized IPTW-weighted rivaroxaban and apixaban cohorts at 3 and 6 months

Outcome	Rivaroxaban, *n* = 1,093, %	Apixaban *n* = 1,344, %	sIPTW, a HR (95% CI)
3 months			
Recurrent VTE or bleeding-related hospitalization	5.3	6.0	0.87 (0.60–1.27)
Recurrent VTE	3.8	4.2	0.92 (0.59–1.42)
Bleeding-related hospitalization	2.4	2.3	1.05 (0.59–1.88)
Critical organ bleed	0.2	0.4	0.49 (0.09–2.59)
Recurrent VTE or critical organ bleed	3.8	4.5	0.85 (0.56–1.31)
6 months			
Recurrent VTE or bleeding-related hospitalization	7.5	7.5	1.00 (0.71–1.40)
Recurrent VTE	5.1	4.9	1.05 (0.71–1.57)
Bleeding-related hospitalization	3.5	3.3	1.06 (0.63–1.79)
Critical organ bleed	0.3	0.7	0.44 (0.13–1.51)
Recurrent VTE or critical organ bleed	5.2	5.3	0.98 (0.66–1.44)

Abbreviations: IPTW, inverse probability of treatment weighting; sIPTW, stabilized inverse probability of treatment weighting; VTE, venous thromboembolism.

a Propensity score model for sIPTW included demographics, laboratory values, clinical observations, comorbidities, cancer type, systemic cancer treatments, and concomitant noncancer medications.

### Falsification Outcome

**Fig. 3:**
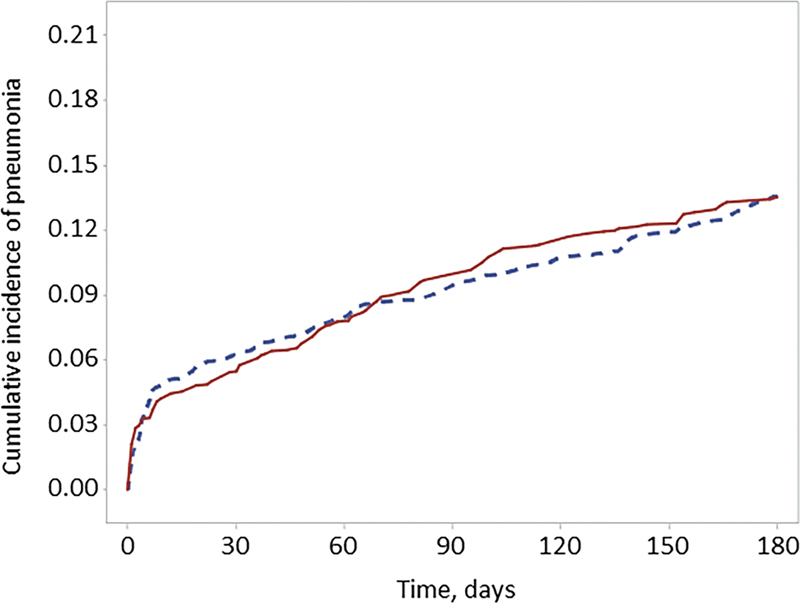
Time to pneumonia (falsification outcome) in rivaroxaban and apixaban patients with Ca-VTE. Kaplan–Meier curve for the falsification outcome of pneumonia (rivaroxaban = solid line, apixaban = dashed line). Ca-VTE, cancer-associated venous thromboembolism.

No statistical difference between the rivaroxaban or apixaban cohorts was observed for our a priori chosen falsification outcome of pneumonia at 3 (8.8 vs. 8.4%; HR: 1.06, 95% CI: 0.75–1.48) or 6 months (11.3 vs. 11.4%; HR: 1.00, 95% CI: 0.75–1.33) ( Fig. 3 ).

## Discussion

To our knowledge, this is the first head-to-head comparative study of rivaroxaban and apixaban for the treatment of Ca-VTE. This was a large study including 2,400+ patients with active cancer, excluding esophageal, gastric, unresected colorectal, bladder, noncerebral central nervous system cancers, and leukemia, and who experienced an incident VTE event. We found no significant difference in the hazard of developing the composite outcome of recurrent VTE or any bleed resulting in hospitalization at 3 months between rivaroxaban- and apixaban-treated patients (HR: 0.87, 95% CI: 0.60–1.27). The HR of 0.87 in combination with an upper limit of the 95% CI of 1.27 (a value less than the 1.5 margin determined to be acceptable a priori 15 ) lends strength to our interpretation that these two oral factor Xa inhibitors have similar effectiveness and safety. The present study also did not find statistically significant differences in the composite outcome of recurrent VTE or any bleed resulting in hospitalization at 6 months, or differences in the composite of recurrent VTE or critical organ bleed, recurrent VTE, bleeding-related hospitalization, or critical organ bleed alone at 3 or 6 months. Finally, the falsification outcome of pneumonia was found to have similar incidence rates between the rivaroxaban and apixaban cohorts, supporting a minimal impact of potential residual confounding bias on our study main findings.

Our results in the present head-to-head comparative study are consistent with the findings of a “living” network meta-analysis (NMA) of RCTs, 19 even after our exclusion of patients with esophageal, gastric, unresected colorectal, bladder, noncerebral central nervous system cancers, and leukemia. In the NMA performed by Riaz and colleagues, 19 no differences were observed in patients' odds of developing recurrent VTE or major bleeding (odds ratio [OR]: 1.10; 95% credible interval [CrI]: 0.56–2.12), recurrent VTE alone (OR: 0.84; 95% CrI: 0.35–1.93), major bleeding alone (OR: 1.57; 95% CrI: 0.54–4.54), or mortality (OR: 0.95; 95% CrI: 0.60–1.49) when comparing rivaroxaban and apixaban (referent). Comparisons of rivaroxaban or apixaban to edoxaban in the NMA also did not show significant differences in these same outcomes with edoxaban. We were unable to assess edoxaban's effectiveness and safety in comparison to rivaroxaban or apixaban in the present study due to edoxaban's low usage in the United States. Future real-world studies comparing edoxaban to apixaban and/or rivaroxaban should be performed in countries where edoxaban has sufficient utilization in the treatment of Ca-VTE.

Multiple medical organizations have published guidance recommending an oral factor Xa inhibitor preferentially over LMWH for the treatment of Ca-VTE in patients with cancer types not associated with a high risk of bleeding when taking an oral factor Xa inhibitor after careful consideration of cancer-specific bleeding risk, potential drug–drug interactions, availability of treatment options, and patient preference. 4 5 6 7 8 9 Patient preference for anticoagulant treatment in VTE appears to favor once-daily intake of oral treatments that do not require dose adjustment or biomonitoring. 20 Rivaroxaban is administered once daily in Ca-VTE after an initial 21 days of twice-daily dosing. In comparison, apixaban is administered twice daily for the entire course of Ca-VTE treatment. Once-daily dosing of direct-acting oral anticoagulants has also been found to be associated with better medication adherence compared to twice-daily dosing in the setting of nonvalvular atrial fibrillation. 21

Since this study utilized a retrospective cohort design and was based on routinely collected data, various biases may have affected our results. 22 Misclassification bias is always a concern in retrospective analyses. We attempted to attenuate this risk by using validated coding algorithms to identify active cancer diagnoses, covariates, and outcomes. The use of an EHR data set also provided us with laboratory and observation values reducing our reliance on billing codes to identify the presence or absence of many key covariates (e.g., BMI, eGFR, anemia, thrombocytopenia, etc.). Moreover, we limited identification of recurrent VTE to the presence of ≥1 of a validated set of VTE-associated billing codes restricted to the primary coding position during an inpatient encounter (previously shown to have a positive predictive value of about 95%). 12 To detect bleeding-related hospitalizations, we used the validated Cunningham algorithm. 13 To address the risk of confounding bias, our analysis used a stabilized IPTW approach to balance many important baseline covariates between rivaroxaban- and apixaban-treated patients. 17 Despite our use of stabilized IPTW, residual confounding bias from unmeasured covariates in nonrandomized studies cannot be fully ruled out, even though the lack of difference observed for our falsification analysis does add some reassurance. As we utilized an EHR data set 11 that did not have corresponding claims data for prescriptions, we were unable to formally assess persistence to rivaroxaban or apixaban. The choice of utilizing the anticoagulant used on day 7 as the “intention-to-treat” index anticoagulant therapy was made to prevent early therapy switching within the first 7 days (i.e., from a heparin to rivaroxaban or apixaban) from impacting our results. Lastly, because we evaluated a U.S. Ca-VTE population without cancer types associated with high risk of bleeding; our results and conclusions are most generalizable to that population. While it may be of clinical interest to compare rivaroxaban versus apixaban in patients with Ca-VTE with specific high-risk bleeding cancer types, available sample sizes were not yet large enough to carry out robust analyses in this study.

## Conclusion

Among adult patients with active cancers, excluding esophageal, gastric, unresected colorectal, bladder, noncerebral central nervous system cancers, and leukemia, and experiencing an acute VTE, rivaroxaban appeared to be at least as effective and safe as apixaban at 3 months. No statistically significant differences were observed in any outcome between the anticoagulant cohorts at 3 or 6 months. Given the similar effectiveness and safety of rivaroxaban and apixaban in Ca-VTE treatment, prescribers should consider patient preference, adherence, and other patient-specific factors when choosing the optimal anticoagulant.

## Notes

**What Is known about This Topic?**

- Cancer patients experiencing venous thromboembolism (Ca-VTE) carry an approximately threefold higher rate of thrombosis recurrence and nearly twice the risk of bleeding during anticoagulation treatment.
- Guidelines recommend oral factor Xa inhibitors over LMWH for the treatment of Ca-VTE in patients with cancer types not associated with a high risk of bleeding when taking an oral factor Xa inhibitor.
- No head-to-head trials comparing rivaroxaban and apixaban for the treatment of Ca-VTE are available.

**What Does This Paper Add?**

- Among patients with active cancers associated with a lower risk of bleeding on an oral factor Xa inhibitor and experiencing an acute VTE, rivaroxaban had similar effectiveness and safety to apixaban at 3 months and 6 months.
- Given the similar effectiveness and safety of rivaroxaban and apixaban in Ca-VTE treatment, this study suggests prescribers consider patient preference, adherence, and other patient-specific factors when choosing optimal anticoagulation therapy.
