# Supplementary material for: Rivaroxaban versus Apixaban for Treatment of Cancer-Associated Venous Thromboembolism in Patients at Lower Risk of Bleeding
Source: TH Open. 2023 Jul 10;7(3):e206–16. doi: 10.1055/s-0043-1770783 (PMC10332896; doi:10.1055/s-0043-1770783)
Supplement: Supplementary file 1 — Supplementary Material [file 10-1055-s-0043-1770783-s23040018.pdf]

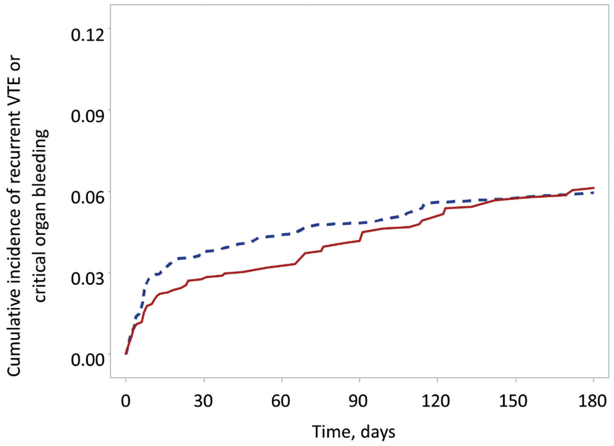


**Supplementary Fig. S1** Time to recurrent venous thromboembolism or critical organ bleeding. Kaplan–Meier curve for recurrent venous thromboembolism or critical organ bleeding (rivaroxaban = solid line, apixaban = dashed line). VTE, venous thromboembolism.

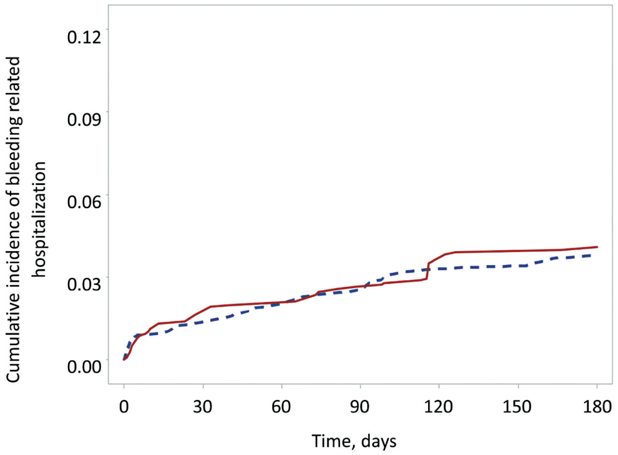


**Supplementary Fig. S3** Time to bleeding related hospitalization. Kaplan–-Meier curve for bleeding related hospitalization (rivaroxaban = solid line, apixaban = dashed line).

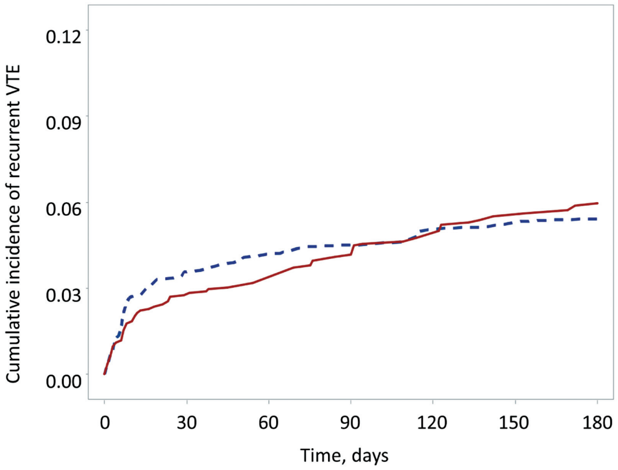


**Supplementary Fig. S2** Time to recurrent venous thromboembolism. Kaplan–Meier curve for recurrent venous thromboembolism (rivaroxaban = solid line, apixaban = dashed line). VTE, venous thromboembolism.

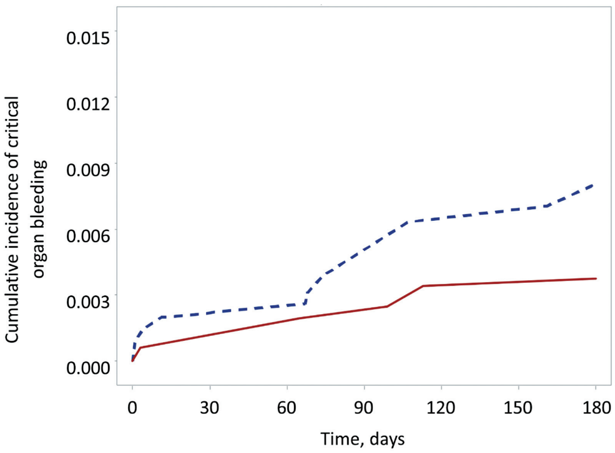


**Supplementary Fig. S4** Time to critical organ bleeding. Kaplan–Meier curve for critical organ bleeding (rivaroxaban = solid line, apixaban = dashed line).

 © 2023. The Author(s).
